# Supplementary material for: The genome sequence of the Horsetail Weevil, Grypus equiseti (Fabricius, 1775) (Coleoptera: Erirhinidae)
Source: Wellcome Open Res. 2026 Apr 22;11:237. [Version 1] doi: 10.12688/wellcomeopenres.26331.1 (PMC13579030; doi:10.12688/wellcomeopenres.26331.1)
Supplement: Peer review history

# Reviewer report: Reviewer response for version 1

**DOI:** 10.21956/wellcomeopenres.28999.r154157

**Published:** 2026-09-15

**Authors:** Eduardo Luís Menezes de Almeida

**Affiliations:** 1 Universidade Federal de Viçosa, Minas Gerais, Brazil

The article presents a genome assembly of *Grypus equiseti. *At the time, it represented the first assembly of the family Erirhinidae. The chromosome-level genome presented is high quality and the manuscript is approved with only two minor points to be addressed:

 Comments: 

 Background:

 I believe readers would appreciate some information, if any are available, on the genetic characteristics of *Grypus *or related organisms to compare with the assembly.

 Methods:

 Why were two different specimens used for genome sequencing and Hi-C sequencing?

Are sufficient details of methods and materials provided to allow replication by others?

Yes

Is the rationale for creating the dataset(s) clearly described?

Yes

Are the datasets clearly presented in a useable and accessible format?

Yes

Are the protocols appropriate and is the work technically sound?

Yes

Reviewer Expertise:

Bioinformatics and computational biology. Microbial genomics and systems biology.

I confirm that I have read this submission and believe that I have an appropriate level of expertise to confirm that it is of an acceptable scientific standard.

# Reviewer report: Reviewer response for version 1

**DOI:** 10.21956/wellcomeopenres.28999.r154150

**Published:** 2026-05-19

**Authors:** Duong Huy Nguyen

**Affiliations:** 1 Vietnam Academy of Science and Technology, Hanoi, Vietnam

Reviewer Report

**Overall Assessment**  This Data Note presents the first chromosome-level reference genome for *Grypus equiseti* (Horsetail Weevil, Erirhinidae), produced through the Darwin Tree of Life (DToL) pipeline. The primary genome assembly has a total length of 779.27 megabases, with 98.69% of the sequence successfully scaffolded into 12 chromosomal pseudomolecules, including the X sex chromosome. The mitochondrial genome was also fully assembled with a length of 19.25 kilobases. The datasets and raw sequencing reads are fully deposited and openly accessible in INSDC databases. The manuscript is well-structured, the experimental design is standard for the DToL consortium, and the resulting genomic resource holds immediate value for comparative coleopteran genomics and biological control monitoring.

**Detailed Comments and Feedback**

 Comment 1: The reported contig N50 is 0.97 Mb, falling marginally below the Earth BioGenome Project (EBP) benchmark of ≥1 Mb, yet this discrepancy is completely unacknowledged and undiscussed in the main text. The authors need to provide a clear explanation in the Results section clarifying whether the manual curation steps involving 79 breaks and 74 joins were the direct cause of this reduced contig contiguity. Furthermore, providing both the pre- and post-curation contig N50 values would allow readers to properly evaluate the trade-off between continuity and structural accuracy.

 Comment 2: The k-mer completeness of the primary assembly is only 83.07%, which is significantly lower than the EBP benchmark of ≥95% and is currently only acknowledged implicitly through Table 4. It is recommended that the authors directly discuss and clarify this issue within the text rather than merely listing it in the table. Specifically, they should analyze whether this shortfall is a consequence of the read distribution driven by the high heterozygosity of 0.91%, as estimated by GenomeScope2, when running Hifiasm in --primary mode.

 Comment 3: The study utilizes two different specimens collected two years apart for the PacBio HiFi sequencing data (icGryEqui2, collected in 2023) and the Hi-C data (icGryEqui1, collected in 2021), but the manuscript provides no discussion on the potential risk of inter-individual structural variations introducing errors during the scaffolding process. The authors should supplement the text with the scientific rationale for using two different specimens, clarify whether they were collected from the same population, and include a statement confirming that inter-individual genomic variation is unlikely to have compromised scaffolding fidelity, as visually supported by the clean Hi-C contact map in Figure 3.

 Comment 4: Although assigning the X sex chromosome based on the mapping of ancestral X BUSCO genes is an appropriate approach, the manuscript lacks crucial technical details necessary for experimental reproducibility, specifically omitting which ancestral X BUSCO reference dataset was used and the exact number of genes successfully mapped. The authors should incorporate these details into the Methods section and clarify whether the resulting synteny pattern clearly differentiates the X chromosome from the autosomes without ambiguity.

 Comment 5: There is a mathematical inconsistency where the authors report a sequencing coverage of "approximately 47×" in the genome sequence report section, whereas a direct calculation from the primary data in Table 1 (37.07 Gb of total data divided by the estimated haploid genome size of 741.29 Mb) yields roughly 50×. The authors should correct this figure to ~50× to align with the provided tables, or explicitly clarify in the text whether the 47× coverage reflects the remaining data after initial read filtering and quality trimming.

 Comment 6: The assembled mitochondrial genome length is reported at 19.25 kb, which is noticeably larger than the typical coleopteran mitogenome range of 15–18 kb, yet the manuscript completely lacks any structural or annotation context to explain or validate this unusual size. To enhance the scientific utility of this Data Note, the authors should add a brief description of the gene content, confirming the presence of the expected 13 protein-coding genes, 22 tRNAs, and 2 rRNAs, and clarify whether this expanded size is driven by an enlarged control region, duplications, or non-coding intergenic spacers.

 Comment 7: Although the European Nucleotide Archive accession identifier PRJEB79531 is listed in the Data Availability section , the corresponding datasets and raw sequence data are currently not accessible or remain private in the INSDC databases. For a Data Note, immediate data availability is the core requirement for indexing; therefore, the authors must either officially release these datasets to the public or provide a secure, private reviewer access key/token so that the quality, formatting, and usability of the raw reads and final assembly can be properly verified before the manuscript is approved.

Are sufficient details of methods and materials provided to allow replication by others?

Yes

Is the rationale for creating the dataset(s) clearly described?

Yes

Are the datasets clearly presented in a useable and accessible format?

No

Are the protocols appropriate and is the work technically sound?

Yes

Reviewer Expertise:

Bioinformatics and comparative genomics, with a specific focus on bacterial and fungal genome analysis, assembly, and microbial diversity.

I confirm that I have read this submission and believe that I have an appropriate level of expertise to confirm that it is of an acceptable scientific standard, however I have significant reservations, as outlined above.

# Reviewer report: Reviewer response for version 1

**DOI:** 10.21956/wellcomeopenres.28999.r154152

**Published:** 2026-05-12

**Authors:** Mohamed Abdelsalam

**Affiliations:** 1 Cairo University, Giza, Egypt

This Data Note reports a chromosome-level genome assembly for *Grypus equiseti* (Horsetail Weevil; Erirhinidae), produced through the Darwin Tree of Life pipeline using PacBio HiFi and Arima Hi-C sequencing. It is the first genome for the genus *Grypus* and for the family Erirhinidae, which has immediate value for comparative coleopteran genomics and biocontrol research. The assembly is technically sound and largely meets EBP standards. Two metrics fall marginally below benchmark, warranting brief comment.

**1. Contig N50 falls just below EBP benchmark.** The reported contig N50 is 0.97 Mb against the EBP benchmark of ≥1 Mb (Table 4; EBP metric 5.C.Q57 vs. benchmark 6.C.Q40). The authors list this accurately in Table 4, but make no mention of it in the narrative. While the shortfall is minimal and the overall assembly quality is clearly high — the BUSCO completeness and QV scores are excellent — the authors should briefly acknowledge this discrepancy and whether the manual curation steps (79 breaks, 74 joins) partially contributed to the lower-than-benchmark contig N50.

**2. Primary k-mer completeness is below EBP benchmark.** The primary assembly k-mer completeness is 83.07%, against the ≥95% benchmark. The authors acknowledge this only implicitly through Table 4. While the combined completeness reaches 98.86%, many downstream users will work from the primary assembly alone. The discrepancy should be noted explicitly and briefly contextualised — for instance, whether this reflects expected heterozygosity-driven read distribution between primary and alternate haplotypes in Hifiasm's --primary mode rather than genuine sequence loss.

- **Two specimens were used** — icGryEqui2 for HiFi sequencing and icGryEqui1 (collected ~2 years earlier) for Hi-C. The authors note both in the Methods, but the implications for Hi-C scaffolding quality are not discussed. Cross-specimen Hi-C is standard practice in DToL, but explicit acknowledgment that inter-individual variation is unlikely to compromise scaffolding (as borne out by the clean contact map) would strengthen reproducibility reporting.
- **The X chromosome assignment** relies on mapping of ancestral X BUSCO genes, which is appropriate. However, the number of BUSCO genes mapped and the synteny source (which ancestral X set) are not stated. A single sentence specifying the reference would aid reproducibility.
- **The typo "Finley Huchinson"** (missing 't') appears in the Methods section (Sample acquisition paragraph) while the author list correctly reads "Hutchinson." This should be corrected.
- **The 47× coverage statement** in the Genome sequence report section appears to be rounded; the actual figure from Table 1 (37.07 Gb / 741.29 Mb estimated genome size) yields ~50×. The authors should clarify or correct this.
- **The mitochondrial genome length of 19.25 kb** is somewhat larger than the typical coleopteran mitogenome (~15–18 kb). This is not necessarily problematic — some weevil mitogenomes are large — but a brief note on gene content or annotation completeness of the mitogenome would be appropriate, particularly given the biocontrol interest in this species.
- **The biocontrol context** mentioned in the Background (New Zealand introduction, 2017) is the most compelling applied motivation for this genome, yet it receives only one sentence. A brief statement on how the genome may specifically inform risk assessment, host specificity analysis, or population genomic monitoring of the introduction would strengthen the ecological relevance of the Note.

Are sufficient details of methods and materials provided to allow replication by others?

Yes

Is the rationale for creating the dataset(s) clearly described?

Yes

Are the datasets clearly presented in a useable and accessible format?

Yes

Are the protocols appropriate and is the work technically sound?

Yes

Reviewer Expertise:

sequencing

I confirm that I have read this submission and believe that I have an appropriate level of expertise to confirm that it is of an acceptable scientific standard.
